# Eosinophilia and clinical outcome of chronic obstructive pulmonary disease: a meta-analysis

**DOI:** 10.1038/s41598-017-13745-x

**Published:** 2017-10-18

**Authors:** Jeffery Ho, Wajia He, Matthew T. V. Chan, Gary Tse, Tong Liu, Sunny H. Wong, Czarina C. H. Leung, Wai T. Wong, Sharon Tsang, Lin Zhang, Rose Y. P Chan, Tony Gin, Joseph Leung, Benson W. M. Lau, William K. K. Wu, Shirley P. C. Ngai

**Affiliations:** 1 Department of Anesthesia and Intensive Care, The Chinese University of Hong Kong, Hong kong Special Administrative Region, China; 2 Department of Rehabilitation Sciences, The Hong Kong Polytechnic University, Hong Kong Special Administrative Region, China; 3 State Key Laboratory of Digestive Disease, LKS Institute of Health Sciences, The Chinese University of Hong Kong, Hong Kong Special Administrative Region, China; 4 Department of Medicine & Therapeutics, The Chinese University of Hong Kong, Hong Kong Special Administrative Region, China; 5 Tianjin Institute of Cardiology, Tianjin Medical University Hospital 2, Tianjin, People’s Republic of China; 6 School of Nursing, Tung Wah College, Hong Kong Special Administrative Region, China

## Abstract

Numerous studies have investigated the association between eosinophilia and clinical outcome of patients with chronic obstructive pulmonary disease (COPD) but the evidence is conflicting. We conducted a pooled analysis of outcome measures comparing eosinophilic and non-eosinophilic COPD patients. We searched articles indexed in four databases using Medical Subject Heading or Title and Abstract words including COAD, COPD, eosinophil, eosinophilia, eosinopenia from inception to December 2016. Observational studies and randomized controlled trials with parallel groups comparing COPD patients with and without eosinophilia were included. Comparing to the non-eosinophilic group, those with eosinophilic COPD had a similar risk for exacerbation in 12 months [Odds ratio = 1.07, 95% confidence interval (CI) 0.86–1.32, *P* = 0.55] and in-hospital mortality [OR = 0.52, 95% CI 0.25–1.07]. Eosinophilia was associated with reduced length of hospital stay (*P* = 0.04). Subsequent to therapeutic interventions, eosinophilic outpatients performed better in pulmonary function tests [Mean Difference = 1.64, 95% CI 0.05–3.23, *P* < 0.001]. Inclusion of hospitalized patients nullified the effect. Improvement of quality of life was observed in eosinophilic subjects [Standardized Mean Difference = 1.83, 95% CI 0.02–3.64, *P* = 0.05], independent of hospitalization status. In conclusion, blood eosinophilia may be predictive of favorable response to steroidal and bronchodilator therapies in patients with stable COPD.

## Introduction

Chronic obstructive pulmonary disease (COPD) is an obstructive airway disease with both overlapping and distinctive features as with asthma^1^. Asthma is characterized by eosinophilic inflammation^2^, whereas COPD is predominantly associated with neutrophilic inflammation in the airways^3^. Growing evidence suggested that neither characteristic was immutably ingrained in either disease. This difference in cellular composition of induced sputum may, if ever, be indistinguishable between these disease groups^2^. Increased sputum eosinophils has been reported in both stable^3^ and exacerbation phase^4^ of patients with COPD, implying the potential role of eosinophils in the pathogenesis of COPD^2^.

Eosinophilia is generally defined as greater or equal to 2% eosinophils in either blood or sputum^3,5–7^. Alternatively, an absolute blood eosinophil count of 0.34 × 10^9^ cells per liter can be used as a threshold for risk stratification^7^. Peripheral blood eosinophil count is highly associated with eosinophilia of the respiratory tract^5^. This blood biomarker has also been shown to reflect submucosal eosinophilia of the lung and reticular basement membrane thickening^8^. Given this context, we considered that patients with COPD who had more than 2% of eosinophils, either in the blood or sputum, as eosinophilic COPD.

Acute exacerbation of COPD significantly increases symptoms, deteriorates pulmonary function, increases rate of hospitalization and lengthens hospital stay further impairing functional capacity and quality of life (QOL) imposing additional burden to healthcare system^9–11^. The in-hospital mortality can reach 30% or more^12^. Seeking for predictive biomarkers for clinical outcome in this population is thus of high priority.

Numerous studies have evaluated eosinophilia in relation to exacerbation risk^5,7,13^, length of hospital stay^14–16^, in-hospital mortality^12,17,18^, and response to steroidal and bronchodilator therapies^9–11^ but the evidence is conflicting. Some studies have reported a higher risk for exacerbation in patients with eosinophilic COPD^13,19^. Conversely, a retrospective study suggested that a higher level of eosinophils protected against disease aggravation^16^. Other research teams failed to detect any association^5,7,20^.

We conducted a systematic review and meta-analysis of clinical outcome measures comparing patients with COPD who had eosinophilia and those without eosinophilia.

## Results

**Figure 1:**
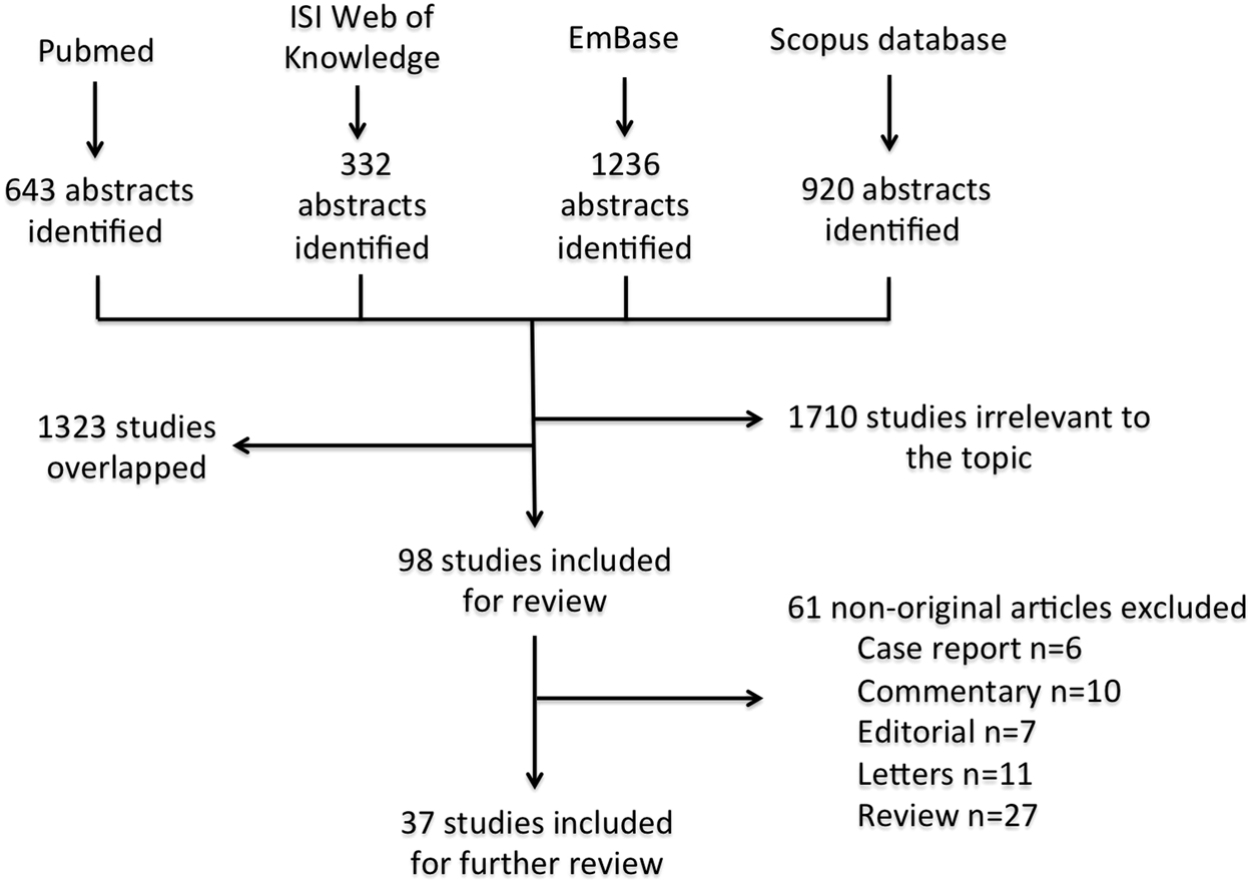
Flow diagram of literature search and selection of studies.

Of 3,131 abstracts identified by the initial search, 1,710 and 1,323 articles were removed, respectively, because of irrelevance or overlaps. After exclusion, 37 studies involving 99,122 patients published between 1998 and 2016 were included for qualitative synthesis (Fig. 1). Of these, 14 studies were included in meta-analysis. The number of entries derived from different search terms has been summarized in Table 1. The mean age of the subjects was 66.95 years with the proportion of male subjects ranging from 45^5^ to 100%^21^. On average, each subject had a 46 pack-year smoking history. The mean forced expiratory volume in one second (FEV_1_) ranged from 0.96 L to 1.62 L. A total of 21 studies explored the role of blood eosinophilia in COPD. The remaining articles detected eosinophils in sputum and bronchial fluid after treatment with bronchodilators or steroidal therapy. The description of studies is summarized in Table 2. More than half of the included studies were either conducted in the United Kingdom^1,9–11,13,17,18,22–27^ or other European countries^2–4,21,28–31^. Eleven studies were originated from the Asia-Pacific region^5,6,32–35^ and the North America^19,20,36–38^. There was only a single relevant publication from the Middle East^12^.

**Table 1:** Number of entries by different search terms.

Keywords	PubMed	ISI	EmBase	Scopus
Eosinophil	41656	19002	53271	—
COPD	66801	36622	59900	—
COAD	62620	406	650	737
Chronic Obstructive Pulmonary Disease	62138	36569	64583	62441
Chronic Obstructive Airway Disease	62957	9182	16818	17754
COPD OR COAD OR Chronic Obstructive Pulmonary Disease OR Chronic Obstructive Airway Disease	68033	53234	91310	75056
(Esosinophil) AND (COPD OR COAD OR Chronic Obstructive Pulmonary Disease OR Chronic Obstructive Airway Disease)	643	332	1236	920
Total	3131

**Table 2:** Description of the included studies.

First author	Year	Country	Single/Multi-center	Number of subjects	Study design	Mean age (Years)	Male (%)	Baseline FEV1	Smoking (Pack-years)	Specimens	Eosinophil measurement
Bafadhel	2009	UK	Single	34	Longitudinal	68	82.4	36% Pred	45	Sputum	Absolute and differential count
Bafadhel	2011	UK	Single	145	Longitudinal	69	70	1.33 L	49	Blood and Sputum	Absolute and differential count
Bafadhel	2012	UK	Single	164	RCT	69	65.2	1.19 L	54.5	Blood and Sputum	Absolute and differential count
Bafadhel	2016	UK	Multiple	243	Prospective cohort	71	55	1.05 L	49	Blood	Absolute and differential count
Balzano	1999	Italy	Single	46	Case-control	66.3	100	46.6% Pred	≥1	Sputum	Differential count and ECP level
Barnes	2016	UK	Single	751	RCT	63.8	72	1.32 L	43.2	Blood	Absolute and differential count
Bathoorn	2009	The Netherlands	Single	45	Longitudinal	64	81.6	63% Pred	40	Blood and Sputum	Absolute and differential count
Brightling	2000	UK	Single	67	RCT	68	59	1.15	33	Sputum	Differential count and ECP level
Couilard	2016	USA	Single	167	Retrospective cohort	71.4	51.5	52.2% Pred	NA	Blood	Differential count
Brightling	2005	UK	Single	60	RCT	67	66	1.22	40	Blood and Sputum	Absolute and differential count
D’Armiento	2009	USA	Single	148	Case-control	65.8	58.1	41.3% Pred	57.8	Lung larvage and plasma	Lung lavage eotaxin-I level
DiSantostefano	2016	USA	Population-based	948	Cross-sectional	59.5	59.7	≤70% Pred	≥10	Blood	Absolute and differential count
Duman	2015	Turkey	Single	1704	Retrospective cohort	70	66.9	≤70% Pred	NA	Blood	Absolute and differential count
Eltobili	2014	USA	Single	103	Case-control	66.5	66.9	51	48	Blood and Sputum	Absolute and differential count
Fabbri	2003	Italy	Single	46	Case-control	65.3	65.2	1.62 L	35.8	Sputum and bronchial biopsy	Differnetial count and histology
Fijimoto	1999	Japan	Single	24	Prospective cohort	69	100	40.5% Pred	60	Sputum	Absolute and differential count
Fujimoto	2005	Japan	Single	62	Longitudinal nested case-control	68.5	94	1.40 L	50.5	Sputum	Absolute and differential count
Gorska	2008	Poland	Single	39	Case-control	56.8	58.8	73% Pred	38.6	Sputum	Absolute and differential count
Hinds	2016	USA	Multiple	3255	RCT	65	61	≤70% Pred	≥10	Blood	Absolute and differential count
Holland	2010	UK	Single	65	Retrospective cohort	75.9	NA	NA	NA	Blood	Differential count
Iqbal	2015	UK	Multiple	4647	Retrospective cohort	≥40	NA	≤70% Pred	≥10	Blood	Absolute and differential count
Kitaguchi	2012	Japan	Single	63	Case-control	72	90.5	47.5% Pred	60.8	Sputum	Absolute and differential count
Louis	2002	UK	Single	49	Case-control	61	73.3	54% Pred	≥20	Sputum	Differential count and ECP level
Mercer	2005	UK	Single	19	Longitudinal	69	85	1 L	NA	Sputum	Absolute and differential count
Negewo	2016	Australia	Multiple	141	Case-control	69.8	63	57.5% Pred	37.5	Blood	Absolute and differential count
Papi	2006	Italy	Single	64	Longitudinal	70.6	87.5	0.96 L	48.3	Sputum	Absolute and differential count
Park	2016	Korea	Single	130	Prospective cohort	67	97.7	≤80% Pred	46	Blood	Absolute and differential count
Pavord	2016	UK	Multiple	3045	Retrospective cohort	64.1	79	≤70% Pred	38	Blood	Absolute and differential count
Perng	2006	Taiwan	Single	62	RCT	72	98.4	1.27 L	48	Sputum	Absolute and differential count
Pesci	1998	Italy	Single	12	Case-control	62.6	91.7	71.1% Pred	38.6	Bronchial larvage	Differnetial count and ECP level
Rahimi-rad	2015	Iran	Single	100	Prospective cohort	70.8	69	37.27% Pred	NA	Blood	Differential count
Salturk	2015	Turkey	Single	647	Retrospective cohort; Nested case-control	68	80.8	NA	41.5	Blood	Differential count
Serafino-Agrusa	2016	Italy	Single	132	Retrospective cohort; Nested case-control	72.9	68.9	44.9% Pred	70.3	Blood	Absolute and differential count
Siva	2007	UK	Single	82	RCT	70	67	1.02 L	49.1	Blood and Sputum	Absolute and differential count
Snoeck-Stroband	2008	The Netherlands	Multiple	114	Case-control	60	86.8	63% Pred	41	Sputum and bronchial biopsy	Absolute and differential count
Vedel-Krogh	2016	Denmark	Population-based	81668	Prospective cohort	58	45	78% Pred	30	Blood	Absolute and differential count
Zanini	2015	Italy	Single	31	Cross-sectional	67	79.3	68% Pred	51	Sputum	Absolute and differential count

Keys: ECP, eosinophil cationic protein; NA, not reported; Pred, predicted; RCT, Randomized controlled trial.

Overall, included studies fell into low to moderate quality (Supplementary Tables 1 and 2). Of 24 non-randomized observational studies evaluated by Newcastle-Ottawa scale, the mean score was 4.5 out of nine (range: 2–6). Five studies scored six or above in a nine-point scale, indicating high study quality^6,7,11,22,30^. In 13 randomized control trials assessed by Cochrane Collaboration Risk of Bias tool, seven studies were rated as low risk in terms of allocation concealment, blinding of participants and personnel, blinding of outcome assessment and incomplete outcome data^9–11,20,24,26,27^. Notably, two studies were ranked as high risk for randomization, blinding, and selective reporting^4,32^.

**Figure 2:**
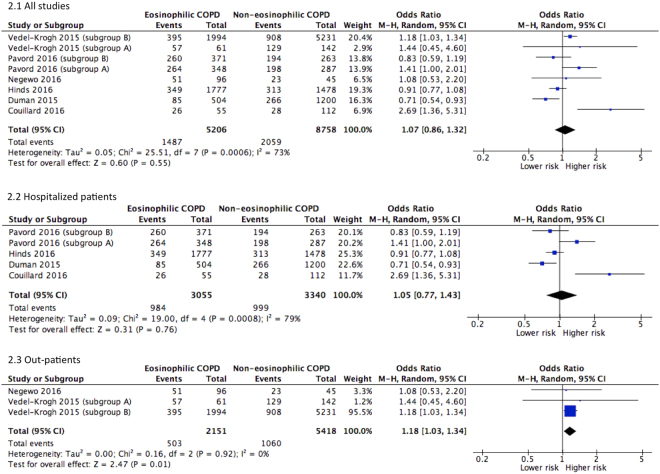
Forest plots of studies comparing the risk for exacerbation in 12 months in COPD patients with or without eosinophilia. Vedel-Krogh (2015) subgroup A, clinical COPD; Vedel-Krogh (2015) subgroup B, COPD cohort in general population; Pavord (2016) subgroup A, COPD patients on fluticasone propionate and salmeterol; Pavord (2016) subgroup B, COPD patients on fluticasone propionate.

Eight populations of six studies^5,7,13,16,19,20^ were pooled for risk analysis. Overall, no association was observed between eosinophilia and risk for exacerbation warranting hospital admission in 12 months (OR = 1.07, 95% CI 0.86–1.32, *P* = 0.55, *I* ^2^ = 73%). This null effect remained in sub-group analysis of studies involving hospitalized COPD patients^13,16,19,20^. Interestingly, in patients with stable COPD as defined as having no hospitalization in the previous 12 months, eosinophilia appears to increase the risk for exacerbation by 18% (OR = 1.18, 95% CI 1.03–1.34, *I* ^2^ = 0%) **(**Fig. 2 **)**.

**Figure 3:**
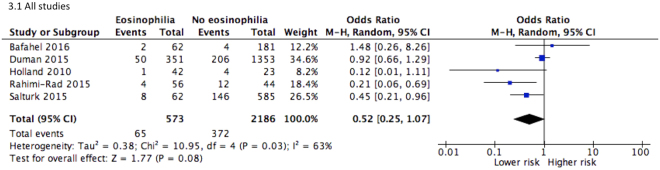
Forest plots of studies comparing the risk for in-hospital mortality in COPD patients with or without eosinophilia.

**Figure 4:**
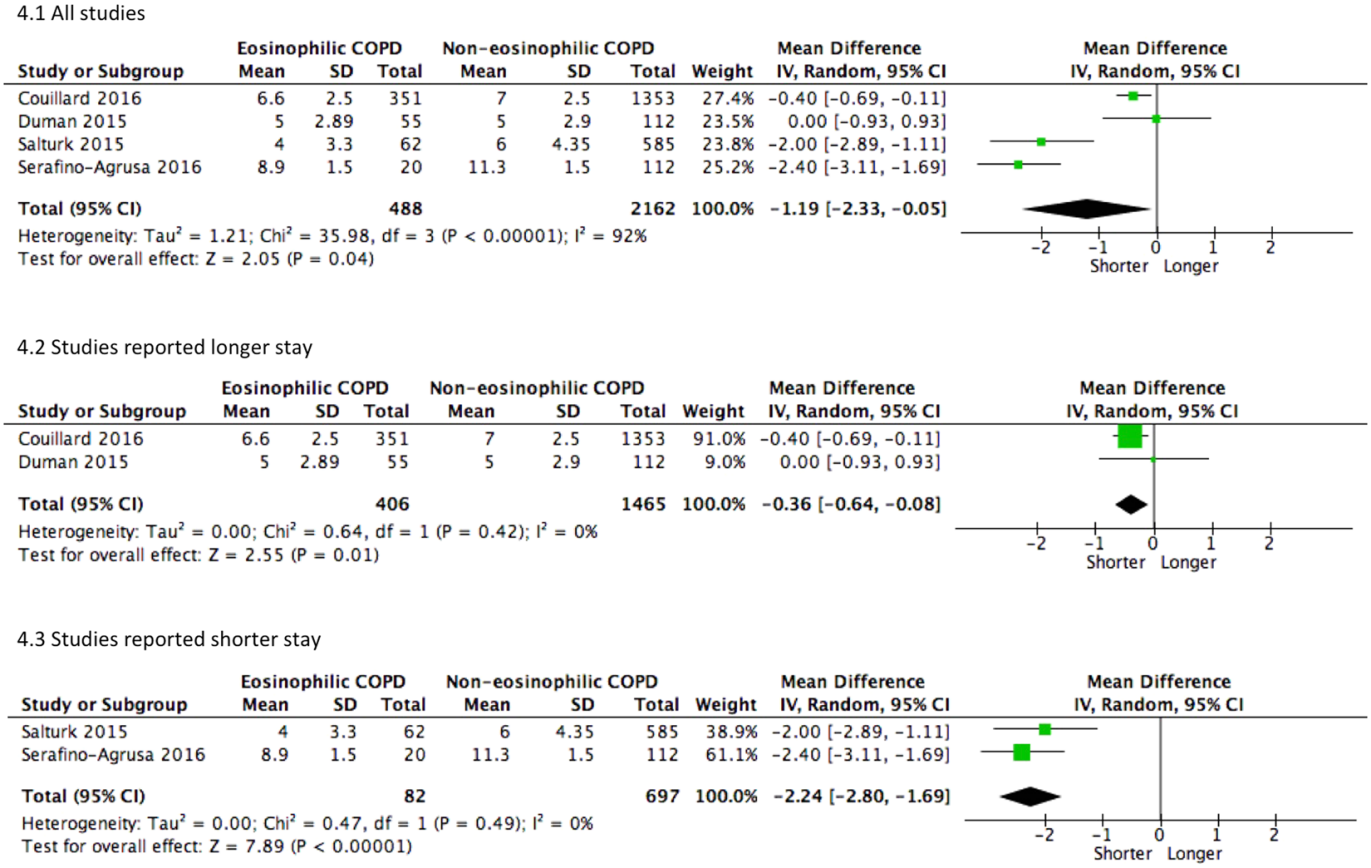
Forest plots of studies comparing the mean difference of the length of hospital stay.

Pooled estimate of five studies^12,14,16–18^ did not indicate an association between eosinophilia and in-hospital mortality, though approaching statistical significance (*P* = 0.08). Of note, a single largest study published in the Lancet^26^ did not identify any association between clinical outcomes and eosinophilia. Although pooled estimate of the other studies^12,14,17,18^ showed that eosinophilia was a protective factor against in-hospital mortality (OR = 0.38, 95% CI 0.17–0.86, *I* ^2^ = 35%), these studies have to be interpreted with cautions due to potential risk of bias. Patients with eosinophilic COPD had 1.2 days shorter hospital stay than non-eosinophilic individuals. Given moderate to high heterogeneity of overall estimates, sensitivity analysis was performed. Except for in-hospital mortality, no single study substantially altered the pooled estimates (Figs 3 and 4).

**Figure 5:**
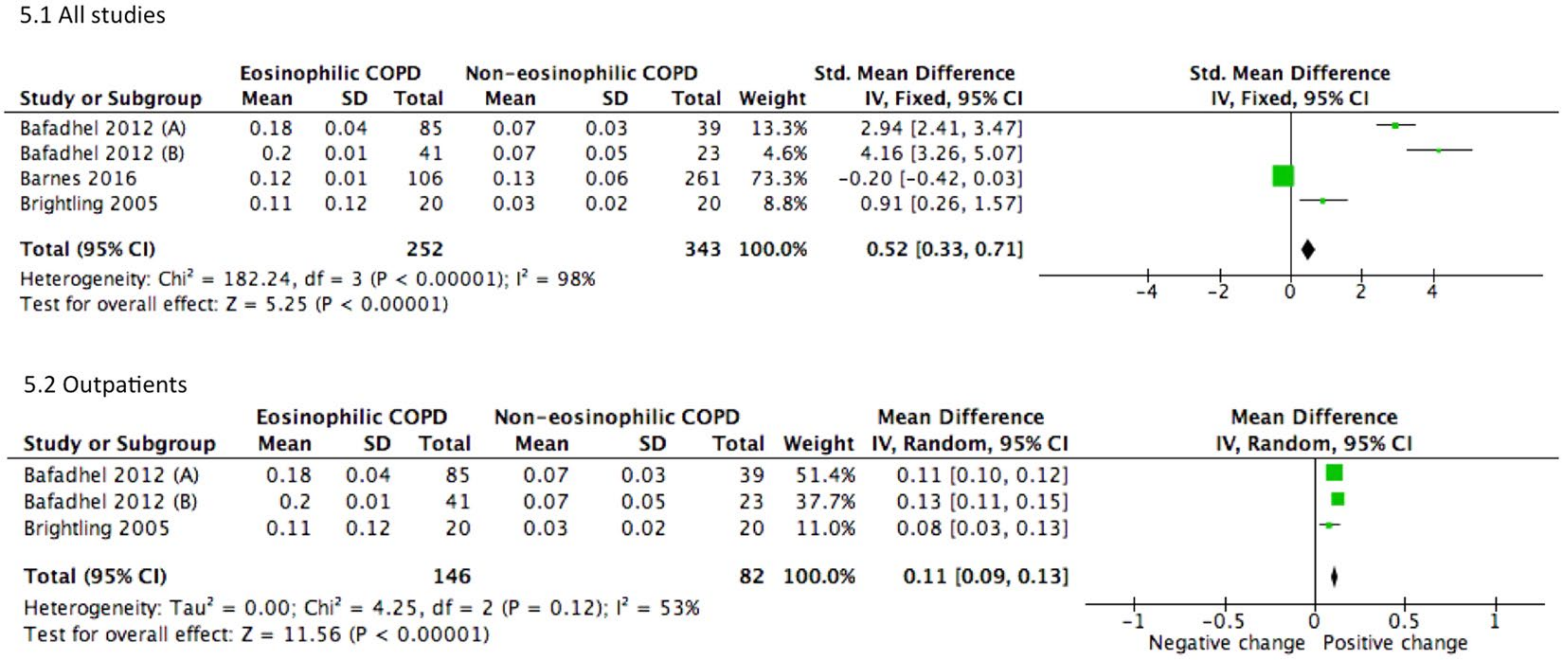
Forest plots of studies comparing the mean difference of the change of FEV1 in COPD patients after therapy. Bafadhel (2012) subgroup A, clinical outcomes in 2 weeks after therapy. Bafadhel (2012) subgroup B, clinical outcomes in 6 weeks after therapy.

**Figure 6:**
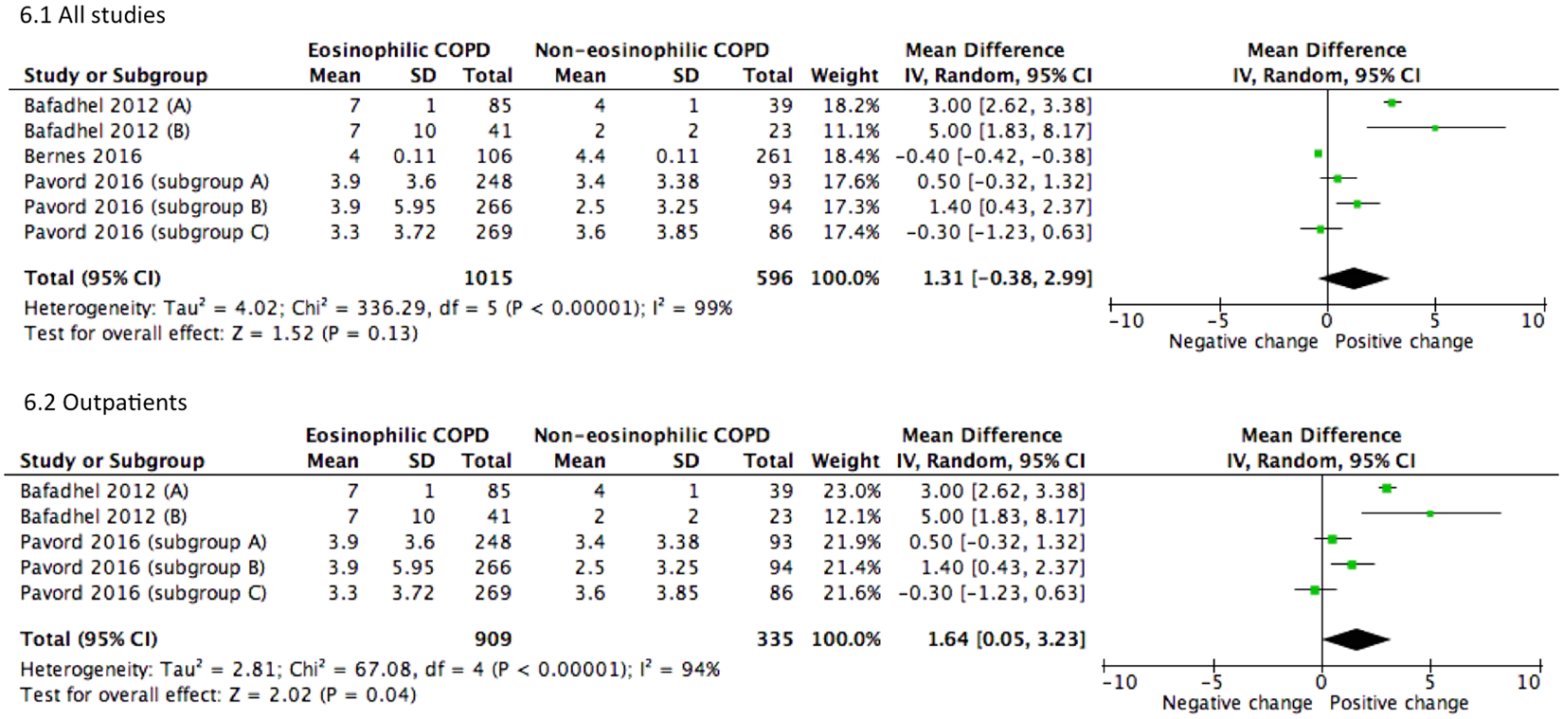
Forest plots of studies comparing the mean difference of the change of % FEV1 predicted in COPD patients after therapy. Bafadhel (2012) subgroup A, clinical outcomes in 2 weeks after therapy. Bafadhel (2012) subgroup B, clinical outcomes in 6 weeks after therapy. Pavord (2016) subgroup A, COPD patients on fluticasone propionate and salmeterol; Pavord (2016) subgroup B, COPD patients on fluticasone propionate; Pavord (2016) subgroup C, COPD patients on salmeterol.

Subsequent to concurrent treatments with bronchodilators and steroids the pooled estimate revealed slight improvement in change of FEV1 (SMD = 0.52, 95% CI 0.33–0.71) (Fig. 5). Sub-group analysis has also shown that outpatients with eosinophilic COPD exhibited improvement in pulmonary function. For outpatient groups, the combined mean differences for FEV_1_ and percentage of predicted FEV_1_ were 0.11 L (95% CI 0.09–0.13, *P* < 0.001) and 1.64% (95% CI 0.05–3.23, *P* < 0.001), respectively (Figs 5 and 6).

**Figure 7:**
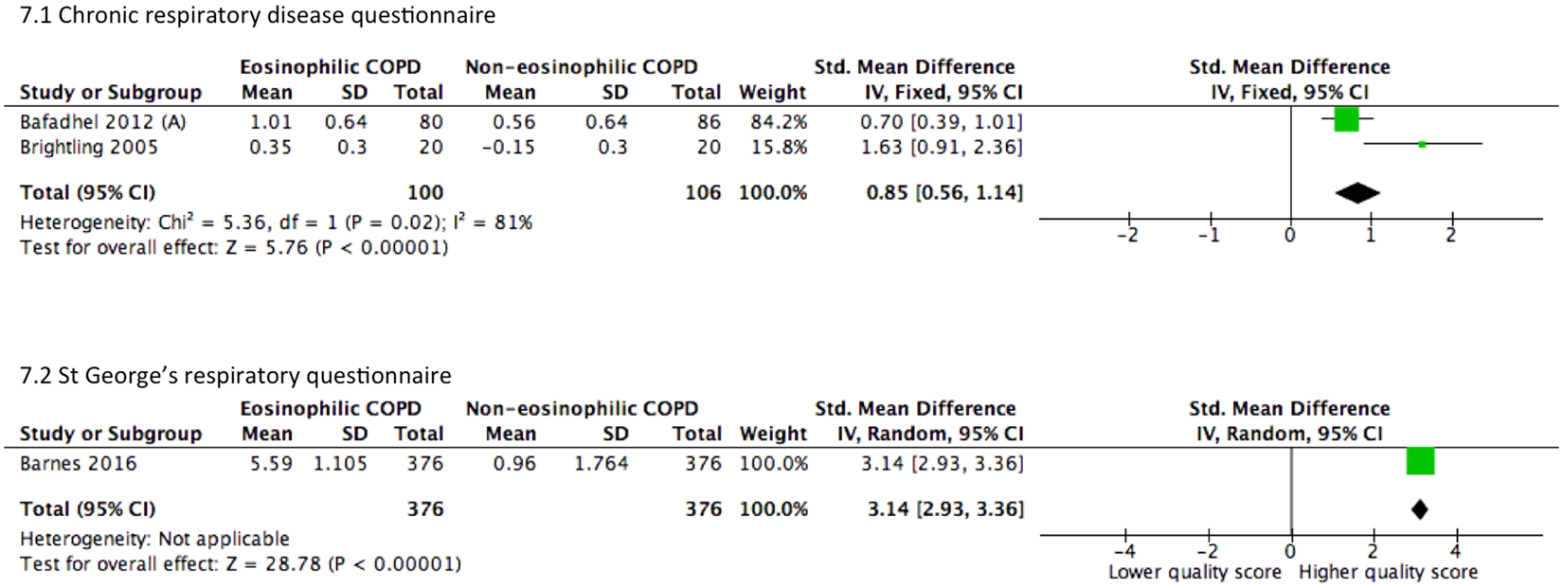
Forest plots of studies comparing the standardized mean difference of the change of quality of life scores in COPD patients after therapy. Pavord (2016) subgroup A, COPD patients on fluticasone propionate and salmeterol.

Of the three studies comparing reported QOL in patients with COPD, chronic respiratory disease questionnaire (CRQ)^9,10^ and St George’s respiratory questionnaire (SGRQ) were used^11^. The eosinophilic group consistently reported a higher QOL score subsequent to therapy. For studies using CRQ, a standardized mean difference of 0.85 (95% CI 0.56–1.14) was observed. For studies using SGRQ, an improved quality of life was also reported (SMD = 3.14, 95% CI 2.93–3.36). The pooled analysis is presented in Fig. 7.

## Discussion

Overall, eosinophilia in COPD patients does not contribute to exacerbation risk, in-hospital mortality, and length of hospital stay. However, higher eosinophil count in the outpatient sub-group demonstrated an increased risk of exacerbation by 18%. On the other hand, eosinophilic COPD patients appeared to be more responsive to therapeutic interventions.

In previous investigation of hospitalized COPD patients with severe exacerbation, eosinophilia lacked association with more than three-fold increased risk for re-admission in 12 months^19^. Retrospective analysis of COPD population with a post-bronchodilator FEV_1_/forced vital capacity (FVC) ratio below 0.7 did not identify significant difference in exacerbation risk amongst the eosinophil dominant group^22^. These were in contrast to a Turkish study in which a greater risk for re-admission was demonstrated in the eosinophilic group^16^. In a Dutch general population study, eosinophilia was found to increase risk for acute exacerbation of COPD^7^. Consistently, we found 18% increased risk for disease aggravation in outpatients. Exacerbation has been linked to airway inflammation characterized by eosinophilia^4,6,24^ and imbalance of metalloproteinases^23^. Higher level of eotaxin, an eosinophil chemotactic factor, is elevated in pulmonary lavage^37^. It has been suggested that frequency and severity of COPD exacerbation was a result of impaired macrophage efferocytosis of eosinophils^36^. Marked eosinophilia was observed in virus-induced exacerbations^30^.

Our pooled analysis showed that eosinophilia is associated with reduced length of hospital stay. This is consistent with previous studies including severely exacerbated COPD patients^14,18^. Conversely, peripheral blood eosinopenia increased in-hospital mortality by up to five-fold^12,17^. The disparity may be attributable to the timing of blood specimen collection. For hospitalized patients, samples were collected at the time of admission^12,14,16–18^. The time for collection in the outpatient group varies across studies and included at the screening stage^11^, at exacerbation^10^, and at 24 h after bronchodilator therapy^9^. In addition, recent hospitalization histories of these outpatients were uncertain^9–11^. In other words, they may have never been hospitalized or had follow-up at clinics soon after discharge. It has been suggested that airway eosinophilia facilitated responsiveness to bronchodilator and steroidal therapies^26,33^. The better response to therapy in this patient population may explain the consistently shorter length of stay and lower mortality.

Eosinophilia has been suggested to indicate individual responsiveness to bronchodilator and steroidal therapies^9–11,13,15,25,26,34^. Post-hoc analysis confirmed that level of eosinophil correlates with the response to bronchodilators^27^. Specifically, post-bronchodilator FEV_1_ and sputum eosinophil level had a high correlation of 0.82^31^. After oral prednisolone therapy, sputum eosinophil count changed accordingly along with interleukin-5^25^. Blood eosinophils were also found to be associated with changes in pulmonary function after inhaled corticosteroids^10,11,13,20^. In our meta-analysis, although the predicted %FEV_1_ changed by 1.64%, this may represent a substantial improvement given these subjects were considered as severe COPD with baseline predicted %FEV_1_ less than 50%^9,10^. However, the addition of hospitalized patients nullified the effect. This suggested that disease severity may be a significant confounder in the observed relationship.

The overall risk of bias in the included randomized control trials ranged from low to moderate. The inferior quality was mostly attributed to unclear sequence generation and likelihood of selective outcome reporting^4,32,34,35,37^. Eight of the studies applied allocation concealment, and blinding of participants and outcome assessors^9–11,18,20,24,27,35^. In quasi-experimental studies, the potential risks of bias included self-reporting for outcomes, insufficient follow-up period and unclear relationship between loss of follow-up and outcome of interest. In addition, appropriate adjustments were not performed for previously reported confounders associated with eosinophil level and clinical outcome of COPD^38^. The majority of the included population was originated from the United Kingdom and other European countries; only a few studies were conducted in the Continent of Asia and the America. This racially skewed population may preclude the generalizability of the evidence.

We performed this systematic review according to a pre-defined data abstraction form. Minor alterations were made to facilitate data pooling. There were missing data on some of the outcome measures of our interest, reducing the number of eligible studies. Given the limited number of included studies for each outcome comparison, neither funnel plot nor Doi plot were conducted to examine publication bias. Our sensitivity analysis revealed that, except for in-hospital mortality, the pooled estimates remained stable.

Given no consensus on definition of eosinophilia, there may be mixing of eosinophilic and non-eoinophilic groups of COPD patient, diluting the effect size. The estimation of eosinophil level varies with the type of specimens. Within the same patient group, bronchial biopsies yielded lower eosinophil count than induced sputum^29^. Importantly, the temporal variation of eosinophilia in COPD was largely ignored in the included studies. Longitudinal study of 1,483 patients with COPD revealed that 49% of the subjects had variable eosinophil counts^39^. Only 37% and 14% of the individuals were persistently eosinophilic and eosinopenic, respectively^39^. The level of this cellular marker can increase considerably soon after sputum induction^40^. In this connection, spotshot sampling may lead to misclassification of case and control.

The moderate to high heterogeneity of the pooled estimates suggests the presence of unknown confounders in association with eosinophilia and COPD. This may be attributed to a range of severity of COPD patients included in the studies and the timing of blood collection. Other potential confounding variables may include, but not limited to, specimen type, baseline characteristics of the study population, study quality and unknown pre-existing co-morbidities. Cross-sectional analysis of 948 COPD patients revealed that eosinophilic group was associated with lower rate of heart attack and anemia^38^. If these contributed to different clinical outcome of this sub-group remained equivocal. The use of steroidal therapy may interfere with the risk for exacerbation. Given the lack of accessibility to information on individual exposure, it was impossible to control for the factor of steroidal therapy in the pooled estimate of exacerbation risk.

In conclusion, eosinophilia is associated with a better improvement of pulmonary function and reported QOL subsequent to therapy in outpatients. Given its association with eosinophil level in the airway, blood eosinophil count may be a predictive biomarker in patients with stable COPD for response to steroidal and bronchodilator therapies.

## Methods

### Searching strategy

This systematic review was performed in accordance with the guidelines on Preferred Reporting Items for Systematic Reviews and Meta-analyses: The PRISMA Statement 2009^41^. Original articles published in PubMed (MEDLINE), ISI Web of Knowledge, EMBASE, and Scopus database were identified using Medical Subject Heading (MeSH) or Title/ Abstract keywords from inception up to December 2016. The MeSH search terms include a combination of eosinophil, blood, sputum, pulmonary disease, chronic obstructive, and/or airway disease. The number of entries retrieved from each database is summarized in Fig. 1. Two authors (JH and WH) performed the literature search and selected the relevant studies independently. Disagreements in terms of study selection were resolved by discussion with senior authors.

### Inclusion and exclusion criteria

Included studies were primary research articles comparing patients with and without eosinophilic COPD in terms of exacerbation risk, mortality, morbidity, length of hospital stay, and response to corticosteroids and bronchodilators. Quasi-experimental studies and randomized controlled trials were included. Pre-clinical studies, review articles, editorials, commentaries, conference abstracts and book chapters were excluded.

### Data extraction

Relevant data were extracted according to a pre-defined data abstraction form. Information on sample size, baseline characteristics, incidence of exacerbation in the past 12 months, length of hospital stay, in-hospital mortality, QOL, and pulmonary function were extracted by one researcher (JH) and verified by a second researcher (WH).

### Quality assessment and statistical analysis

The methodological quality of the included randomized controlled trials and quasi-experimental studies was evaluated by the Cochrane Risk of Bias Tool^42^ and the Newcastle-Ottawa scale^43^ respectively. The former tool indicates studies with high, low or unclear risk according to five domains: selection bias, performance bias, detection bias, attrition bias, and reporting bias. The latter scale evaluates the quality of studies in three attributes, namely selection of cohort, comparability, and outcome. In this review, a high-quality study is defined as having >6 points whereas a low-quality study as having ≤5 points.

Meta-analysis compared patients with eosinophilic and non-eosinophilic COPD in terms of exacerbation risk, length of hospital stay, in-hospital mortality, and change of pulmonary function and QOL in response to medical interventions. Heterogeneity across studies was determined by the *I* ^2^ statistic using Cochrane Review Manager 5.3^44^. An *I* ^2^ values ≥ 25, 50 and 75% were considered as mild, moderate, and high degree of heterogeneity, respectively. For pooled outcome measures with *I* ^2^ > 50%, a random-effect model was used to evaluate the overall effect of a given comparison. Studies were weighted by inverse of variance. Categorical data was presented as odds ratio (OR) in 95% confidence interval (CI). For continuous variables, the pooled estimates were compared by mean difference (MD) or standardized mean difference (SMD), as appropriate. In the occasion when the remaining studies appeared to be different from the overall estimate, sub-group analysis was performed.

## Electronic supplementary material

Supplementary Table 1 and 2
