# Supplementary material for: Eosinophilia and clinical outcome of chronic obstructive pulmonary disease: a meta-analysis
Source: Sci Rep. 2017 Oct 18;7:13451. doi: 10.1038/s41598-017-13745-x (PMC5647332; doi:10.1038/s41598-017-13745-x)
Supplement: Supplementary file 1 — Supplementary Table 1 and 2 [file 41598_2017_13745_MOESM1_ESM.pdf]

Eosinophilia and clinical outcome of chronic obstructive pulmonary disease: a meta-analysis

Jeffery Ho, PhD[1,*], Wangjia He, MSc[2,*], Matthew T.V. Chan, PhD[1], Gary Tse, PhD[4], Tong Liu, PhD[5], Sunny H. Wong, DPhil[3,4], Czarina C.H. Leung, MBChB[1], Wai T. Wong, MBBS[1], Sharon Tsang, PhD[1], Lin Zhang, PhD[1], Rose YP Chan, PhD [6], Tony Gin, MD[1], Joseph Leung, BSc[2], Benson W.M. Lau, PhD[2], William K.K. Wu, PhD[1,3], Shirley P.C. Ngai, PhD[2]

Supplementary Table 1. Risk of bias assessment of the included randomized controlled trials.

	Random sequence generation	Allocation concealment	Blinding of participants	Blinding of outcome assessment	Incomplete outcome data	Selective reporting
Bafadhel 2012	+	?	+	?	+	+
Barnes 2016	?	-	?	?	+	+
Bathoorn 2009	?	+	?	?	?	?
Brightling 2005	+	?	+	-	+	?
Brightling 2000	+	+	+	-	+	?
D'Armiento 2009	+	?	?	+	?	+
Fujimoto 1999	?	?	?	+	+	?
Hinds 2016	?	+	+	+	+	?
Iqbal 2015	?	+	+	+	+	-
Park 2016	?	+	+	+	?	?
Perng 2006	?	?	-	?	+	?
Siva 2007	?	+	+	?	+	+

(+: low risk; -: high-risk; ?: unclear risk)

Supplementary Table 2. Quality assessment of the included non-randomized observational studies.

Frist Author	Study design	**Selection**	**Comparability**	**Outcome**
Bafadhel, 2011	Longitudinal	**	**	**
Bafadhel, 2009	Longitudinal	**	**	
Balzano, 1999	Case-control	**	**	*
Counilard, 2016	Retrospective cohort		**	
DiSantostefano, 2016	Cross-sectional	**	**	
Duman, 2015	Retrospective cohort	*	**	**
Eltobili, 2014	Case-control	**	**	
Fabbri, 2003	Case-control	**	**	
Fujimoto, 2005	Case-control	*	**	**
Gorska, 2008	Case-control	*	**	
Holland, 2010	Retrospective cohort	*	**	
Kitaguchi, 2012	Case-control	**	**	
Louis, 2002	Case-control	*	**	
Mercer, 2005	Longitudinal	*	**	
Negewo, 2016	Case-control		**	
Papi, 2006	Longitudinal	*	**	***
Pavord, 2016	Retrospective cohort	*	**	**
Pesci, 1998	Case-control	**	**	
Rahimi-rad, 2015	Prospective cohort	**	**	*
Salturk, 2015	Retrospective cohort	**	**	
Serafino-Agrusa, 2016	Retrospective cohort	**	**	
Snoeck-Stroband, 2008	Case-control	*	**	
Vedel-Krogh, 2016	Prospective cohort	**	**	**
Zanini, 2015	Cross-sectional	*	**	

Maximum number of stars assigned for each category: Selection (4 stars), Comparability (2 stars), Outcome (3 stars).
